# Comments on ‘dilemma of sigmoid volvulus management’

**DOI:** 10.1308/rcsann.2023.0014

**Published:** 2023-07-12

**Authors:** SS Atamanalp, R Peksoz, E Disci

**Affiliations:** Ataturk University, Turkey

Dear editor,

We read the article written by Abdelrahim *et al*^1^ on the management of sigmoid volvulus (SV). Although SV is a rare clinical entity worldwide, our practising area, Eastern Anatolia, is one of the most important endemic regions for SV. Consequently, our SV series including 1,051 cases treated over a 56-year period (from June 1966 to July 2022) is the largest single-center SV series over the world.^2^ In the light of this experience, our comments relate to the management of SV.

First, the authors reported a recurrence rate of 80% in their 40-case series. In this series, when compared with those of endoscopic decompression alone (33–70%) and emergency surgery (25–50%), the mortality rate of elective surgery was 0% in an 8-patient group, which is significantly lower than that in all American Society of Anesthesiologists (ASA) groups.^1^ In our series, nonoperative decompression was tried in 770 patients (73.3%), whereas emergency surgery was required in 483 cases (46.0%) and elective surgery was used in 115 patients (10.9%). The mortality rates in our three groups, endoscopic decompression alone, emergency surgery and elective surgery, were 0.6%, 17.2% and 0.0%, respectively, whereas early recurrence rates (during the first hospitalization) were 5.5%, 0.6% and 0.0%, and late recurrence rates (in anamnesis or in a mean 20.5-year follow-up period) were 25.8%, 4.1% and 0.0%. Similar to the authors’ and our results, following a successful endoscopic decompression, most literature data currently support the necessity of an elective sigmoid colectomy in selected cases.^3^ However, the actual dilemma is not only treatment protocol but also patient selection. Although elective surgery is generally recommended in nonelderly and well-conditioned patients with SV, patient selection criteria are not identified objectively.^3^ Contrary to the authors’ practice, elective surgery in group ASA 4, in our opinion and experience, patients in ASA groups ≤3, in whom expected operative mortality rate is less than 4.3%, are potential candidates for elective surgery, whereas we recommend percutaneous endoscopic colostomy in patients with ASA groups ≥4 and over 70–75 years of age, whose expected operative mortality rate is higher than 7.8%.^4^

Second, laparoscopic sigmoid colectomy, or even natural orifice specimen extraction (NOSE), are actual minimally invasive procedures that are proposed by most authors.^5^ In our series, 20 of 115 cases (17.4%) were recently treated with laparoscopic surgery, while NOSE will be one of our new targets.

We congratulate the authors for their informative article and await their reply to our comments.
